# *COL4A5* Intronic Variants at Third to Fifth Nucleotides Cause Alport Syndrome

**DOI:** 10.1016/j.ekir.2024.11.016

**Published:** 2024-11-16

**Authors:** Hideaki Kitakado, Tomoko Horinouchi, Shuhei Aoyama, Yuka Kimura, Yuta Inoki, Yu Tanaka, Chika Ueda, Yuya Aoto, Nana Sakakibara, China Nagano, Tomohiko Yamamura, Shingo Ishimori, Rini Rossanti, Masafumi Matsuo, Kandai Nozu

**Affiliations:** 1 Department of Pediatrics, Kobe University Graduate School of Medicine, Kobe, Japan; 2 Department of Pediatrics, Hyogo Prefectural Harima-Himeji General Hospital Center, Himeji, Japan; 3 Department of Child Health, Nephrology Division, Dr. Hasan Sadikin General Hospital/Faculty of Medicine, Universitas Padjadjaran, Bandung, West Java, Indonesia; 4 Graduate School of Science, Technology and Innovation, Kobe University, Kobe, Japan

**Keywords:** Alport syndrome, *COL4A5*, inherited kidney disease, intronic variant, minigene assay, splicing

## Abstract

**Introduction:**

Alport syndrome (AS) is an inherited kidney disease caused by variants in the *COL4A3*, *COL4A4,* or *COL4A5* genes, resulting in type IV collagen abnormalities. Although autosomal dominant variants in *COL4A3* and *COL4A4* are increasingly being diagnosed, X-linked AS (XLAS) caused by *COL4A5* variants predominates. Single nucleotide substitutions in introns positioned at first and second from the last nucleotide (called a consensus sequence) of exons always cause aberrant splicing. However, whether intronic variants at the third to fifth positions from the last nucleotide of exons can cause aberrant splicing is unclear.

**Methods:**

We identified 11 intronic variants positioned at the third, fourth, and fifth nucleotides from the exon 3′ end in *COL4A5* from our AS cohort (January 2006–July 2022). We conducted *in vitro* splicing assays using minigenes and *in silico* splicing analysis using commercial splicing prediction software and evaluated mRNA sequences obtained from patients’ samples when available.

**Results:**

All 11 patients showed aberrant splicing patterns in the minigene splicing assays. *In vivo* analysis of 6 patients corroborated these findings. The commercial splicing prediction software accurately predicted splicing changes in 10 variants.

**Conclusions:**

This study shows that 11 intronic variants at the third to fifth positions in *COL4A5* introns cause aberrant splicing. This finding highlights the importance of evaluating such variants for the diagnosis and prognosis of XLAS. Further investigation is warranted to confirm the pathogenicity of these variants and their effect on the prognosis of the kidney in XLAS.

## Graphical abstract

**Figure:**
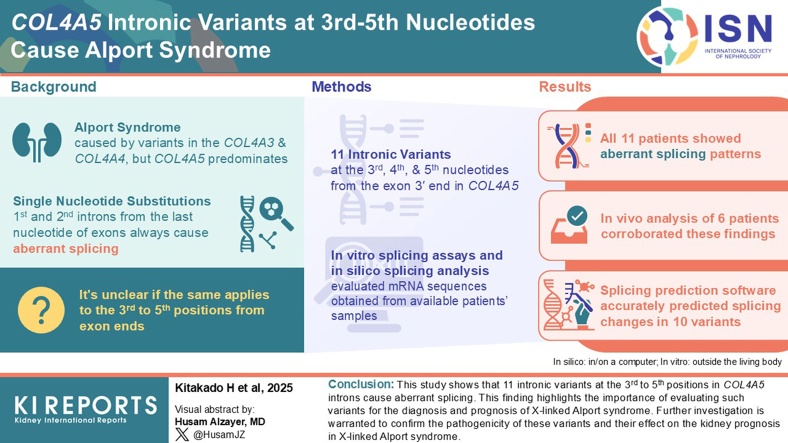

AS is an inherited kidney disease caused by pathogenic variants in *COL4A3*, *COL4A4,* or *COL4A5*, which encode type IV collagen.^1^ Recent advances in technology of genetic analysis have led to an increase in the number of cases diagnosed with autosomal dominant AS caused by variants in *COL4A3* or *COL4A4*. However, we previously reported that 74% of these cases were XLAS caused by variants in *COL4A5.*^2^

XLAS results in a range of kidney symptoms, from only hematuria and/or proteinuria to end-stage kidney disease accompanied by hearing impairment and ocular abnormalities.^3^ Male patients with XLAS typically show a clear genotype-phenotype correlation.^4^, ^5^, ^6^ Patients with truncating variants show the most severe early-onset end-stage kidney disease, those with splicing variants have a moderate phenotype, and those with missense variants have a mild phenotype.^4^, ^5^, ^6^ We previously reported that in our cohort, patients with nonsense variants, splicing variants, and missense variants developed end-stage kidney disease at a median age of 18, 25, and 40 years, respectively.^4^ Treatment with renin–angiotensin–aldosterone system inhibitors, especially in patients with missense variants, improves prognosis of the kidneys.^4^ Therefore, in XLAS, a genetically accurate diagnosis is useful for predicting the prognosis and treatment decisions.

However, as comprehensive genetic analysis becomes more common, evaluation of the pathogenicity of variants is often challenging because of the large number of variants. One of the most important factors in evaluating the pathogenicity of variants is aberrant splicing. We have recently reported that most of the exonic variants positioned first to third from the last nucleotide in the *COL4A5* gene caused aberrant splicing.^7^^,^^8^ Regarding intronic variants, single nucleotide substitutions in introns positioned at first and second from the last nucleotide of exons, which is called a “consensus sequence,” always cause aberrant splicing. However, whether intronic variants positioned at third to fifth from the last nucleotide of exons can cause aberrant splicing is unclear.

## Methods

### Variants

**Figure 1:**
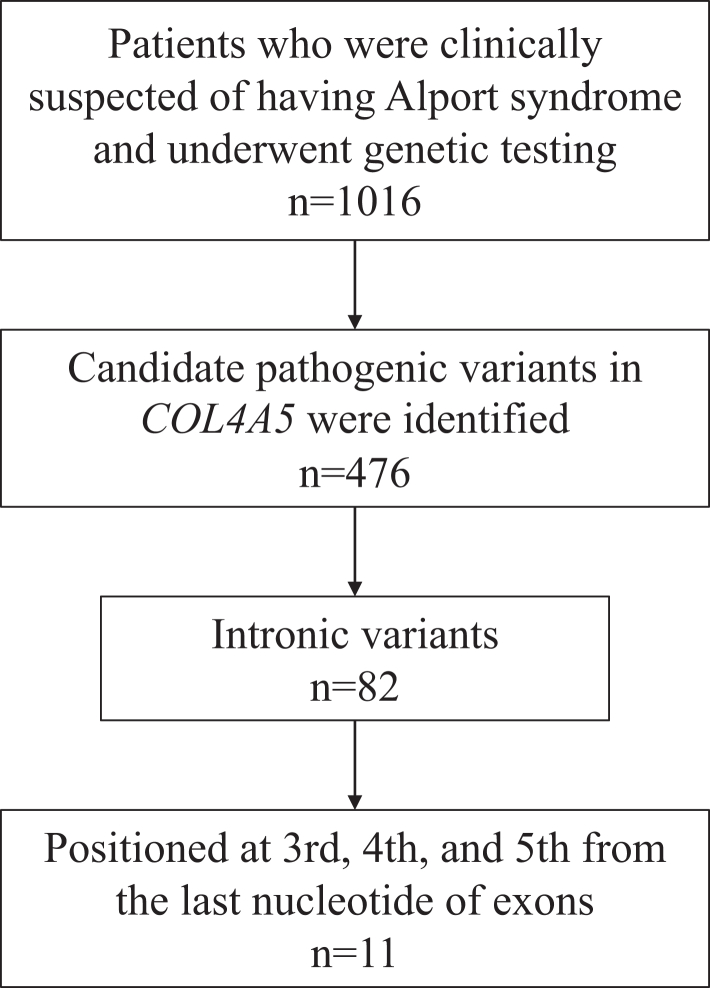
Flow diagram of selecting variants for this study. Among patients clinically suspected of Alport syndrome who underwent genetic testing, 11 with intronic single nucleotide variants (SNVs) positioned third to fifth from the last nucleotide in *COL4A5* were selected for this study.

We extracted all 11 intronic variants positioned at third, fourth, and fifth from the last nucleotide of exons in *COL4A5* from 11 patients in our AS cohort from January 2006 to July 2022 (Figure 1). Our AS cohort included patients in whom clinicians diagnosed AS clinically and requested a genetic analysis at our institution, and in whom a variant that may be associated with disease was identified. Of the 11 intronic variants, 3 had been reported previously from our group.^9^

### Indications for Genetic Testing

Initially, all 11 patients were suspected of AS clinically and referred to have genetic testing. All of them had a kidney biopsy conducted to investigate the cause of hematuria and/or proteinuria. All the patients met at least 1 of the following criteria: (i) a thin basement membrane, stratification, and/or reticular changes observed on electron microscopy; (ii) negative alpha 5 staining (in male patients) or a mosaic defect (in female patients); and (iii) a family history of kidney dysfunction or already diagnosed with AS.

In Case 5, AS was suspected because of persistent hematuria or proteinuria, along with an episode of gross hematuria and findings of thin basement membrane in the kidney biopsy. However, her family history was not available because she had been abandoned by her parents.

### Genetic Analysis With Next-Generation Sequencing

We conducted a genetic analysis as previously reported.^4^^,^^7^^,^^8^ Briefly, genomic DNA was extracted from peripheral blood leukocytes obtained from patients using the QuickGene-Mini80 System or QuickGene-Auto 12S (Kurabo Industries Ltd., Tokyo, Japan) according to the manufacturer’s instructions. Some patients had variants identified through direct sequencing of *COL4A5*. To perform next-generation sequencing, library preparation was conducted using the HaloPlex Target Enrichment System Kit (Agilent Technologies, Santa Clara, CA) by following the manufacturer’s instructions. *COL4A3, COL4A4, COL4A5*, and other podocyte-related genes were sequenced using the MiSeq next-generation sequencing platform (Illumina, San Diego, CA). Sequenced data were aligned to the reference human genome (GRCh37/Hg19) and analyzed with SureCall 4.2.1.10 (Agilent Technologies, CA).

### *In Vitro* Splicing Assay - Minigene Analysis

To construct the hybrid minigene, we used the previously developed H492 vector.^7^^,^^8^^,^^10^ This vector was based on the cDNA 3.0 mammalian expression vector and includes a multicloning site (Invitrogen, Carlsbad, CA) between exons A and B. Using genomic DNA from the wild type and all 11 patients, fragments containing an exon immediately upstream and flanking introns and/or exons were created. To perform cloning, we used the Infusion HD Cloning Kit (Takara Bio Inc., Tokyo, Japan) by following the manufacturer’s instructions.^7^ The constructs were transfected into Hela cells and HEK293T cells using Lipofectamine 2000 (Thermo Fisher Scientific, Tokyo, Japan). Twenty-four hours later, total RNA was extracted from the cells using the RNeasy Plus Mini Kit (Qiagen, Hilden, Germany). We reverse transcribed 1 μg of total RNA using RNA to cDNA EcoDry Premix (Double Primed; Takara Bio Inc.), and polymerase chain reactions were run using a forward primer complementary to a segment upstream of exon A and a reverse primer complementary to a segment downstream of exon B to amplify transcripts from each minigene. The polymerase chain reaction products were analyzed by electrophoresis on a 1.5% agarose gel, followed by Sanger sequencing.

### mRNA Sequencing

Regarding variants of cases 1, 3, 8, 9, 10, and 11, fresh blood samples were available, and mRNA analysis was conducted. Total RNA was extracted from blood leukocytes using the RiboPure Blood Kit (Invitrogen) and an RNA stabilization agent (RNA later; Invitrogen). Total RNA (1–2 μg) was converted to cDNA via reverse transcription with EcoDry Premix (Double Primed; Takara Bio Inc.) and analyzed by polymerase chain reactions amplification.

### *In silico* Splice Site Analysis

We assessed the potential of splicing defects in the variants using SpliceAI (https://spliceai lookup.broad institute.org/). SpliceAI is a deep learning-based tool to predict splice site variants and their potential effect on RNA splicing on a web interface, and the cutoff was set to a Δ score > 0.2.^7^^,^^8^^,^^11^

## Results

A total of 1016 patients had genetic analysis conducted at our department for suspected AS between January 2006 and July 2022. Of the 476 patients with genetically suspected AS, 11 variants within the intron located at +3 to +5 of the exon 3′ end of the *COL4A5* gene were included (Figure 1). All 11 patients’ clinical features are shown in Table 1. All patients had hematuria, and at least 9 of them had proteinuria. All 11 patients have undergone kidney biopsy, and 6 of them had alpha-5 staining performed. Of these, 2 males (case 8 and 10) were completely negative, whereas the remaining 4, including 3 females, were positive. Among 11 patients, only 2 had kidney dysfunction. Moreover, 2 patients had deafness and 1 had amblyopia as extrakidney complications (Table 1). The frequencies of the variants’ positions were as follows; the third position in 3 patients, fourth in 2 patients, and fifth in 6 patients (Table 2).^12^, ^13^, ^14^ Three of these variants had been previously reported.

**Table 1:** Patients’ characteristics

Case	Patients' ID	Age (yr)	Sex	HU	PU (g/gCr)	Cr-eGFR (ml/min per 1.73 m^2^)	Complications	GBM findings in electron microscopy		Alpha 5 staining	Family history
								Thin basement membrane	Basket-weave pattern		
1	121	17	M	+	1.50	127	-	+	-	Positive	Mo, ESKD, 28 yr
2	866	4	F	+	0.21	119	-	+	ND	ND	-
3	876	50	F	+	0.46	68	-	-	+	ND	Fa, ESKD, 37 yr
4	785	23	F	+	ND	ND	-	ND	ND	Positive	Mo, ESKD, 50s
5	635	9	F	+	0.18	132	-	+	-	Positive	ND
6	932	16	M	+	0.55	99	deafness	ND	ND	ND	Mo, ESKD, 30 yr
7	684	29	M	+	2.45	19	-	+	-	ND	Mo/Bro, HU
8	615	3	M	+	0.15	141	amblyopia	+	+	Negative	-
9	402	7	M	+	0.19	98	-	+	+	ND	-
10	904	13	M	+	0.47	116	deafness	ND	ND	Negative	-
11	452	14	F	+	0.36	110	-	+	+	Positive	-
Total	-	NA	NA	11/11	NA	NA	3/11	7/8	4/7	2/6	5/10
Median	-	14	NA	NA	0.41	113	NA	NA	NA	NA	NA

Bro, brother; Cr-eGFR, creatinine-based estimated glomerular filtration rate; ESKD, end-stage kidney disease; F, female; Fa, father; GBM, glomerular basement membrane; HU, hematuria; M, male; Mo, mother; NA, not applicable; ND, not determined; PU, proteinuria.

**Table 2:** *In vitro* and *in vivo* results

Case	Intron	Variant	Transcription (*in vitro*)	Transcription (*in vivo*)	Reference
1	9	c.546+2dupT	exon9 skipping	exon9 skipping	Groopman *et al.*^12^
2	9	c.546+2_3insTT	exon9 skipping	NA	
3	12	c.687+5G>C	exon12 skipping	exon12 skipping	
4	12	c.687+5G>A	exon12 skipping	NA	Al-Dewik *et al.*^13^
5	14	c.834+5G>T	exon14 skipping	NA	Wang *et al.*^14^
6	16	c.936+4A>G	exon16 skipping	NA	
7	16	c.936+5delG	exon16 skipping	NA	
8	18	c.1032+5G>C	exon18 skipping	exon18 skipping	
9	27	c.2146+4delT	exon27 skipping	exon27 skipping	
10	29	c.2395+3A>G	exon29 skipping	exon29 skipping	
11	29	c.2395+5G>A	exon29 skipping	exon29 skipping	

NA, not available.

All 11 variants showed aberrant splicing patterns that were detected by the minigene analysis (Table 2).^12^, ^13^, ^14^ Of the 11 intronic variants, 3 had been reported previously,^9^ and in both cases, mRNA analysis showed that exon skipping was induced. All variants showed aberrant splicing with entire exons skipped compared with wild type results (Table 2,^12^, ^13^, ^14^ Supplementary data). In addition, in the *in vivo* analysis, aberrant splicing patterns were identical to the minigene analysis results in all 6 variants (cases 1, 3, 8, 9, 10, and 11) for which mRNA sequences were available (Table 2).^12^, ^13^, ^14^ The *in silico* analysis also showed a Δ score of SpliceAI > 0.2 in 10 of 11 variants (Table 3). Therefore, we concluded that all 11 variants had pathogenicity because of aberrant splicing.

**Table 3:** *In silico* results

Case	Intron	Number of bases in the preceding exon	Variant	SpliceAI			
				(Δ score of donor loss)	Position (bp)	(Δ score of acceptor loss)	Position (bp)
1	9	81	c.546+2dupT	0.42	−1	0.34	−81
2	9	81	c.546+2dupTT	0.42	−1	0.34	−81
3	12	42	c.687+5G>C	0.52	−5	0.55	−46
4	12	42	c.687+5G>A	0.52	−5	0.55	−46
5	14	54	c.834+5G>T	0.50	−5	0.55	−58
6	16	45	c.936+4A>G	0.23	−4	0.15	−48
7	16	45	c.936+5delG	0.98	−4	0.42	−48
8	18	42	c.1032+5G>C	0.88	−5	0.84	−46
9	27	105	c.2146+4delT	0.06	−3	0.04	−107
10	29	151	c.2395+3A>G	0.72	−3	0.73	−153
11	29	151	c.2395+5G>A	0.37	−5	0.39	−155

## Discussion

In this study, all patients who were clinically diagnosed with AS and had suspected variants within the intron located at +3 to +5 of the exon 3′ end of *COL4A5* showed aberrant splicing. This finding suggested their pathogenic significance. We have already reported that exonic variants positioned at first to third to the last nucleotides in the *COL4A5* gene cause aberrant splicing.^7^^,^^8^ Therefore, single nucleotide substitutions around the end of exons may be pathogenic owing to associated aberrant splicing and need to be carefully evaluated.

Several studies have shown a genotype-phenotype correlation in male patients with XLAS.^4^, ^5^, ^6^ In male patients with XLAS, truncating variants have a worse prognosis than nontruncating variants. In addition, patients with XLAS and truncating splicing variants have a worse prognosis of the kidney than those with nontruncating splicing variants. We previously found that *COL4A5* gene splice site variants with an in-frame deletion at the transcript level showed good prognosis of the kidney, and end-stage kidney disease developed 9 years later than in those with out-of-frame deletion.^9^ These results emphasize the importance of investigating not only the presence of aberrant splicing but also the specific splicing patterns.

The most accurate and reliable method for evaluating splicing is to examine transcripts from kidney tissue; however, obtaining kidney samples is challenging. Therefore, analyzing samples from peripheral blood lymphocytes may offer an alternative. However, obtaining a sufficient quantity and quality of mRNA from blood samples is also difficult. Indeed, in our previous report on XLAS caused by substitutions at the last nucleotide of the exon, an mRNA analysis of blood samples was not available in any of the 20 patients.^7^ To address this issue, we have established an *in vitro* splicing analysis system using minigenes and reported its utility.^7^^,^^8^^,^^15^, ^16^, ^17^, ^18^ In this study, we used the minigene splicing assay to demonstrate that substitutions, deletions, or insertions at the third, fourth, and fifth positions in *COL4A5* introns caused aberrant splicing of entire exon skipping in all 11 cases. Furthermore, all *in vivo* transcript analyses of 6 variants (cases 1, 3, 8, 9, 10, and 11) using patients’ blood samples showed the same splicing pattern. These results suggest that variants in these positions in the *COL4A5* gene are likely to cause aberrant splicing, and the minigene splicing assay could help confirm the effect of variants on aberrant splicing.

In our analysis, SpliceAI accurately predicted aberrant splicing in 10 of the variants (except for case 9). SpliceAI is an *in silico* analysis tool that uses a deep learning model.^11^^,^^19^ This tool calculates the difference in predicted probabilities (ranging from 0 to 1) between wild type and mutant sequences at the splice acceptor and splice donor sites, displaying the highest value as the Δ score.^11^ A Δ score ≥ 0.2 is considered significant.^11^ Splicing is greatly influenced by the surrounding sequence. Therefore, SpliceAI analyzes a region encompassing 500 nucleotides upstream and downstream of the variant (total of 1001 nucleotides).^11^ We analyzed our cohort of 11 patients using SpliceAI. Acceptor loss (removal of the 5′ end of an exon) was predicted in 9 of 11 cases, and donor loss (removal of the 3′ end of an exon) was predicted in 10 of 11 cases. Although predicting more complex splicing patterns might be challenging, SpliceAI is considered highly useful as a complementary tool for *in vivo* or *in vitro* analyses.

Our study has some limitations. First, we only analyzed 11 cases, mixed with male and female patients. Second, among the 11 patients, only 2 had kidney dysfunction. In addition, only 2 patients had hearing loss and 1 patient had amblyopia as extrakidney complications. Therefore, the association between aberrant splicing and the clinical course could not be fully verified. However, these mild phenotypes are thought to be caused by skipping of entire exons, most of which consist of bases with multiples of 3, resulting in an in-frame transcript. Third, whereas the available results of mRNA analysis of the patients’ samples were completely consistent with those of the minigene splicing assay results, mRNA analysis was available in only 6 of the 11 patients.

## Conclusion

This study shows that substitutions, deletions, or insertions at positions third, fourth, and fifth in *COL4A5* introns cause aberrant splicing, leading to relatively mild phenotypes. Therefore, when substitutions, deletions, or insertions at these positions in each *COL4A5* intron are detected, they can be pathogenic and can predict prognosis of the kidney. Further analysis should be performed to confirm whether these variants can cause XLAS by aberrant splicing.

## Disclosure

KN is a member of advisory groups for Kyowa Kirin Co. Ltd., Toa Eiyo Ltd., Zenyaku Kogyo Co. Ltd., and Taisho Pharmaceutical Co. Ltd; has a patent for developing exon skipping therapy for patients with Alport syndrome; and received lecture fees from Ono Pharma, Astellas Pharma, Novo Nordisk Pharma, Alexion Pharma, Sumitomo Pharma, Sanofi, Otsuka Pharma, Daiichi Sankyo, and Miyarisan. All the other authors declared no competing interests.

## Supplementary Material

Supplementary File (PDF) **Supplementary Data.** Results of *in vitro* analysis of 11 patients.

## Footnotes

Supplementary File (PDF)
**Supplementary Data.** Results of *in vitro* analysis of 11 patients.
