# Supplementary material for: COL4A5 Intronic Variants at Third to Fifth Nucleotides Cause Alport Syndrome
Source: Kidney Int Rep. 2024 Nov 16;10(2):516–21. doi: 10.1016/j.ekir.2024.11.016 (PMC11843108; doi:10.1016/j.ekir.2024.11.016)
Supplement: Supplementary File (PDF) — Supplementary Data. Results of in vitro analysis of 11 patients. [file mmc1.pdf]

COL4A5 intronic variants at 3rd-5th nucleotides cause Alport syndrome

Hideaki Kitakado, MD [1)], Tomoko Horinouchi, MD, PhD[1)], Shuhei Aoyama, MD[1)], Yuka Kimura, MD [1)], Yuta Inoki, MD [1)], Yu Tanaka, MD [1)], Chika Ueda, MD [1)], Yuya Aoto, MD, PhD [1, 2)], Nana Sakakibara, MD, PhD [1)], China Nagano, MD, PhD [1)], Tomohiko Yamamura, MD, PhD [1)], Shingo Ishimori, MD, PhD [1)], Rini Rossanti, MD,PhD[1), 3)], Masafumi Matsuo[4)], Kandai Nozu, MD, PhD [1)]

1) Department of Pediatrics, Kobe University Graduate School of Medicine, Kobe, Japan
2) Department of Pediatrics, Hyogo Prefectural Harima-Himeji General Hospital Center, Himeji, Japan
3) Department of Child Health, Nephrology Division, Dr. Hasan Sadikin General Hospital/Faculty of Medicine, Universitas Padjadjaran, Bandung, West Java, Indonesia
4) Graduate School of Science, Technology and Innovation, Kobe University, Kobe, Japan

Corresponding author

Tomoko Horinouchi, MD, PhD

Department of Pediatrics, Kobe University Graduate School of Medicine,

7-5-1 Kusunoki-cho, Chuo, Kobe, Hyogo 650-0017, Japan

E-mail: tohori@med.kobe-u.ac.jp

Tel: +81-78-382-5111

Fax: +81-78-382-5050

ORCID: https://orcid.org/0000-0003-1655-6030

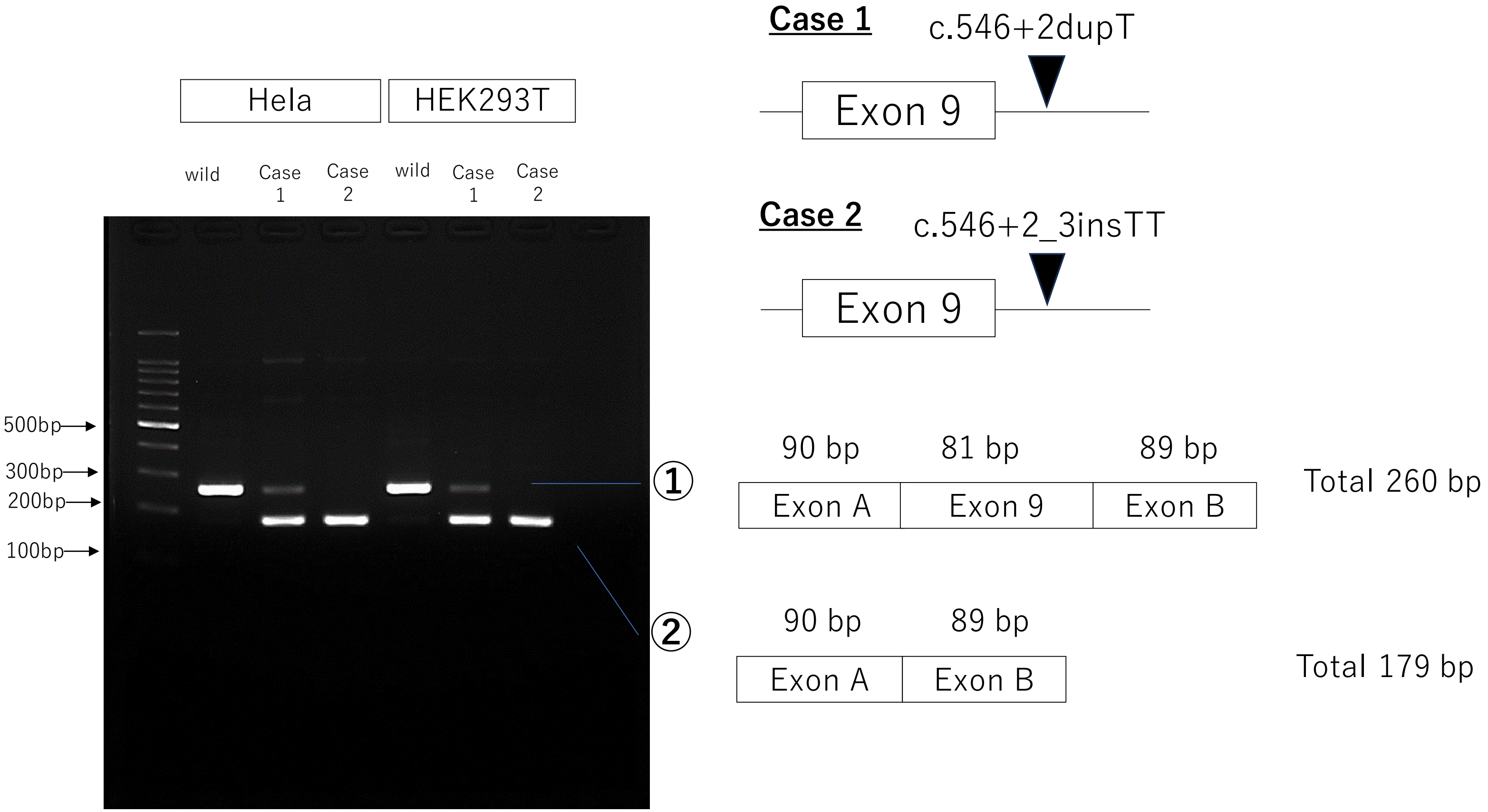
Hela
HEK293T
wild
Case 1
Case 2
wild
Case 1
Case 2
500bp
300bp
200bp
100bp
①
②
Case 1
c.546+2dupT
Exon 9
Case 2
c.546+2_3insTT
Exon 9
90 bp
81 bp
89 bp
Exon A
Exon 9
Exon B
Total 260 bp
90 bp
89 bp
Exon A
Exon B
Total 179 bp

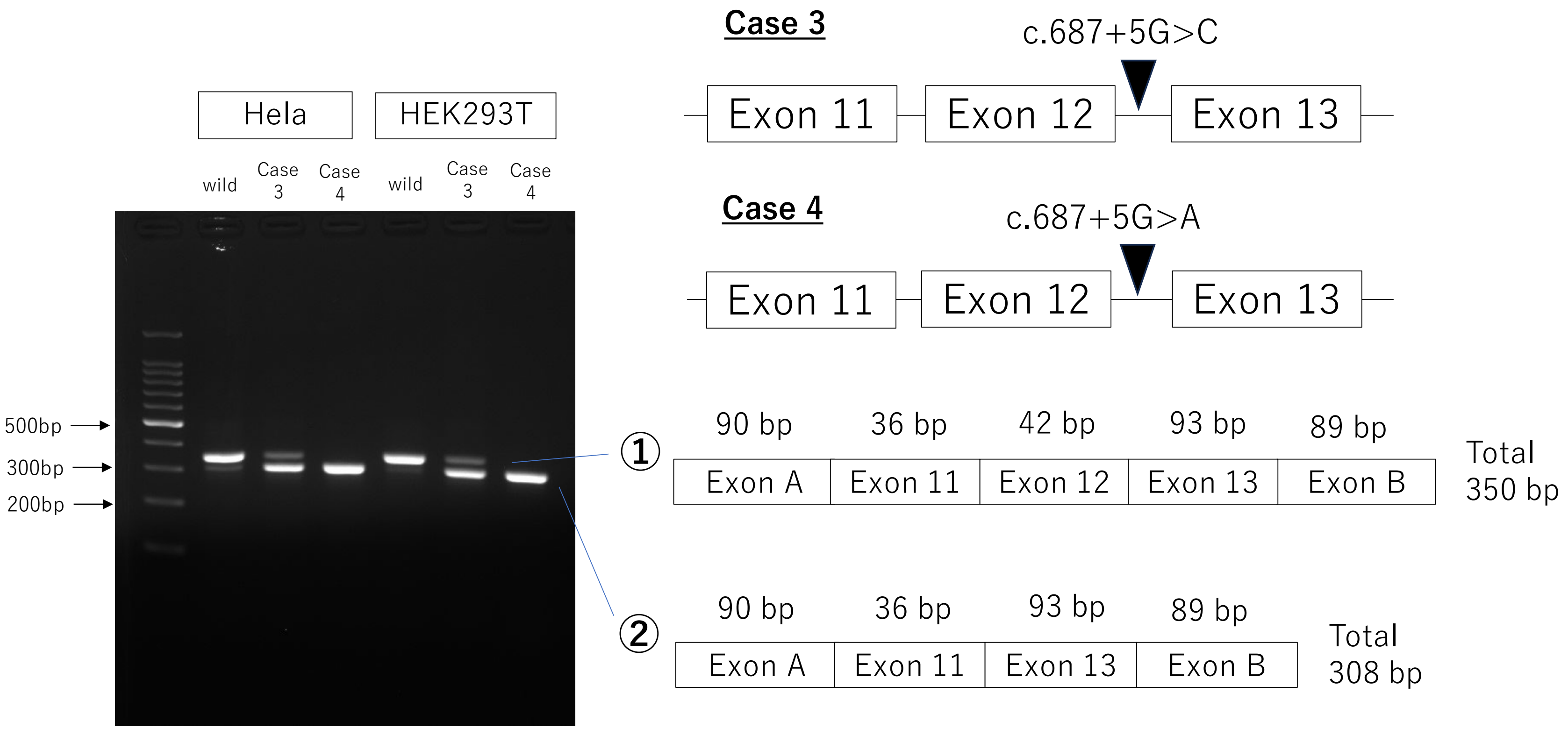
Hela
HEK293T
wild
Case 3
Case 4
wild
Case 3
Case 4
500bp
300bp
200bp
Case 3
c.687+5G>C
Exon 11
Exon 12
Exon 13
Case 4
c.687+5G>A
Exon 11
Exon 12
Exon 13
①
90 bp
36 bp
42 bp
93 bp
89 bp
Exon A
Exon 11
Exon 12
Exon 13
Exon B
Total 350 bp
②
90 bp
36 bp
93 bp
89 bp
Exon A
Exon 11
Exon 13
Exon B
Total 308 bp

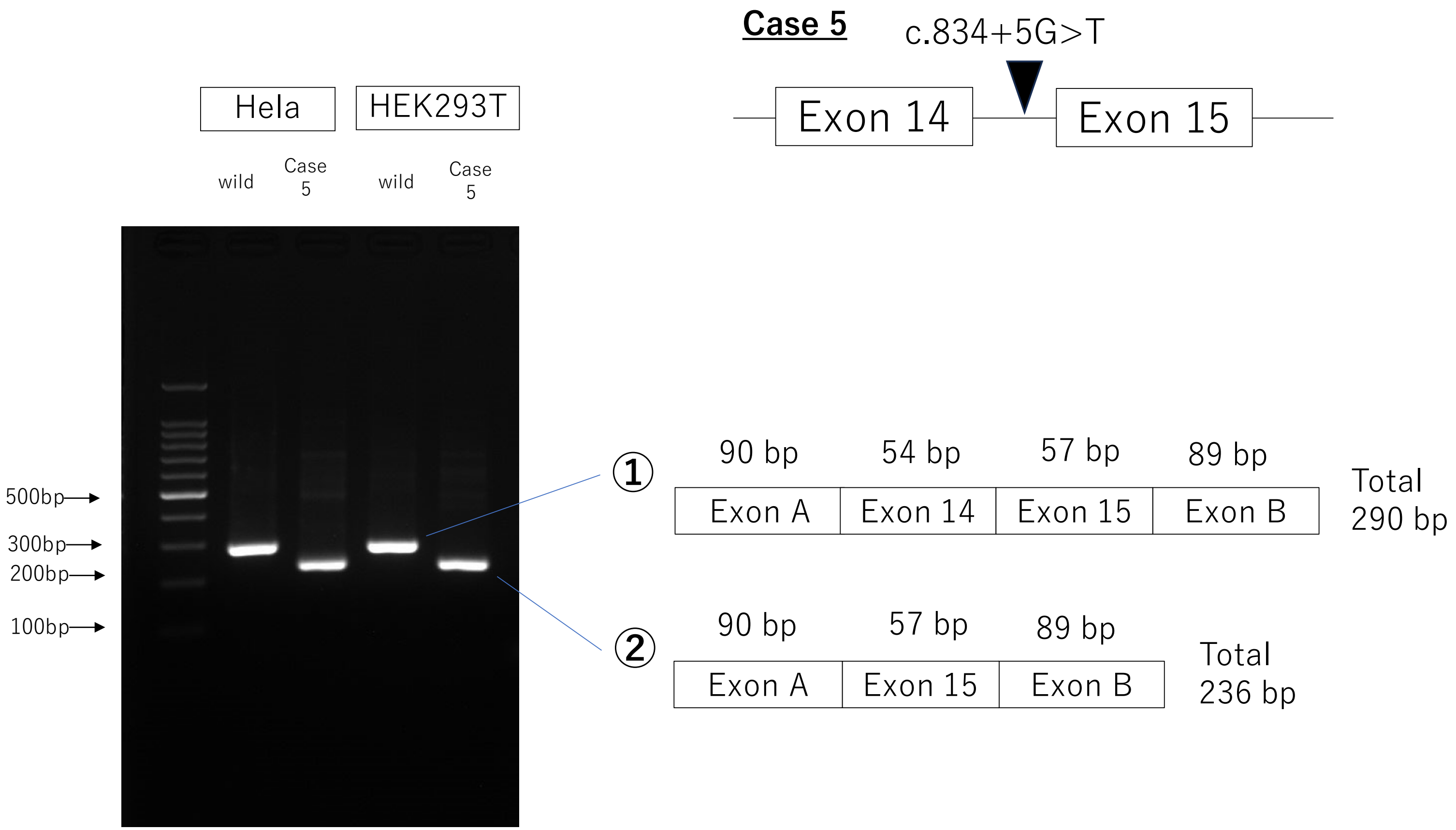
Case 5
c.834+5G>T
Exon 14
Exon 15
Hela
HEK293T
wild
Case 5
wild
Case 5
500bp
300bp
200bp
100bp
①
90 bp
54 bp
57 bp
89 bp
Exon A
Exon 14
Exon 15
Exon B
Total 290 bp
②
90 bp
57 bp
89 bp
Exon A
Exon 15
Exon B
Total 236 bp

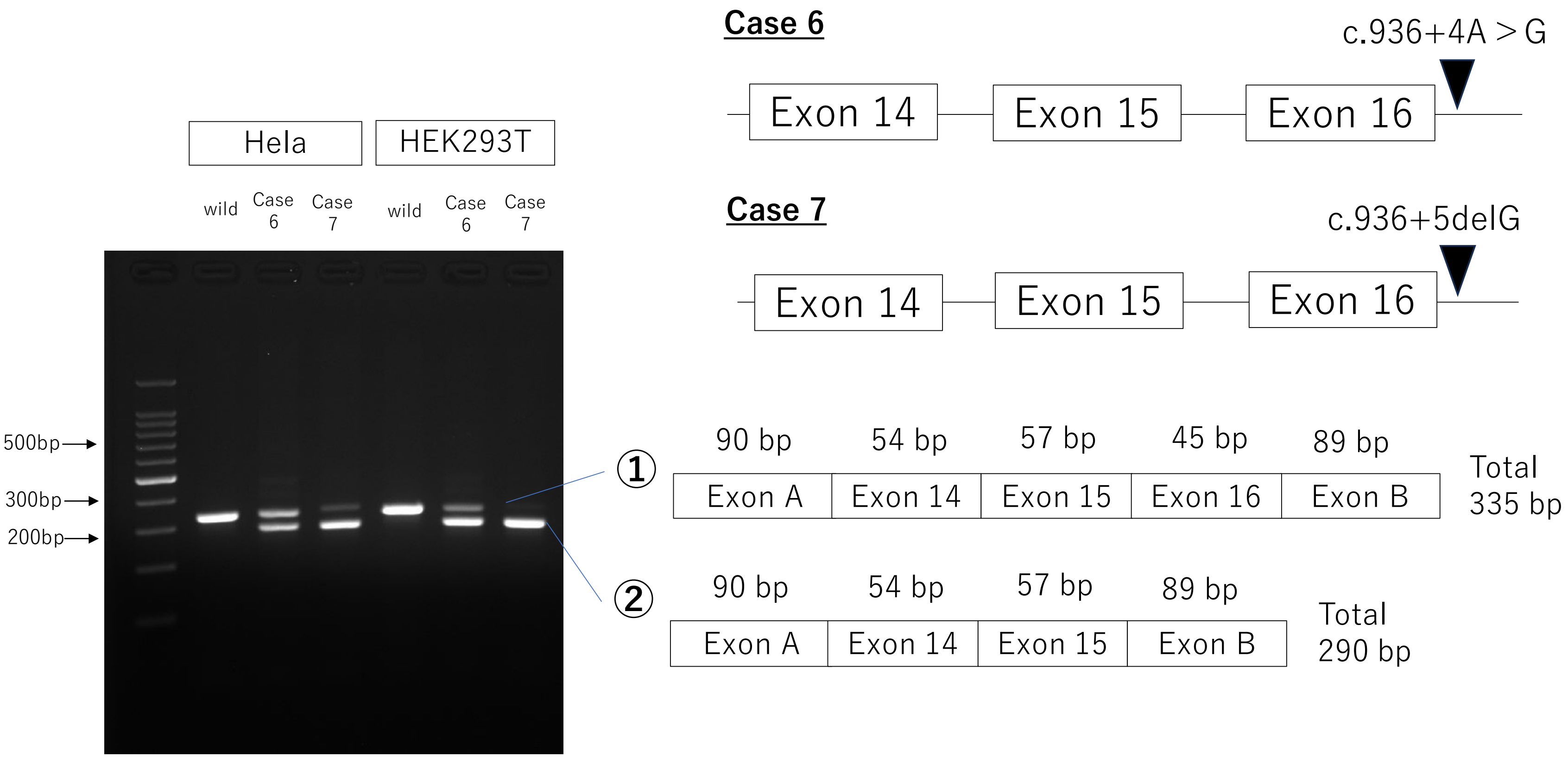
Hela
HEK293T
wild
Case 6
Case 7
wild
Case 6
Case 7
500bp
300bp
200bp
Case 6
c.936+4A＞G
Exon 14
Exon 15
Exon 16
Case 7
c.936+5delG
Exon 14
Exon 15
Exon 16
①
90 bp
54 bp
57 bp
45 bp
89 bp
Exon A
Exon 14
Exon 15
Exon 16
Exon B
Total 335 bp
②
90 bp
54 bp
57 bp
89 bp
Exon A
Exon 14
Exon 15
Exon B
Total 290 bp

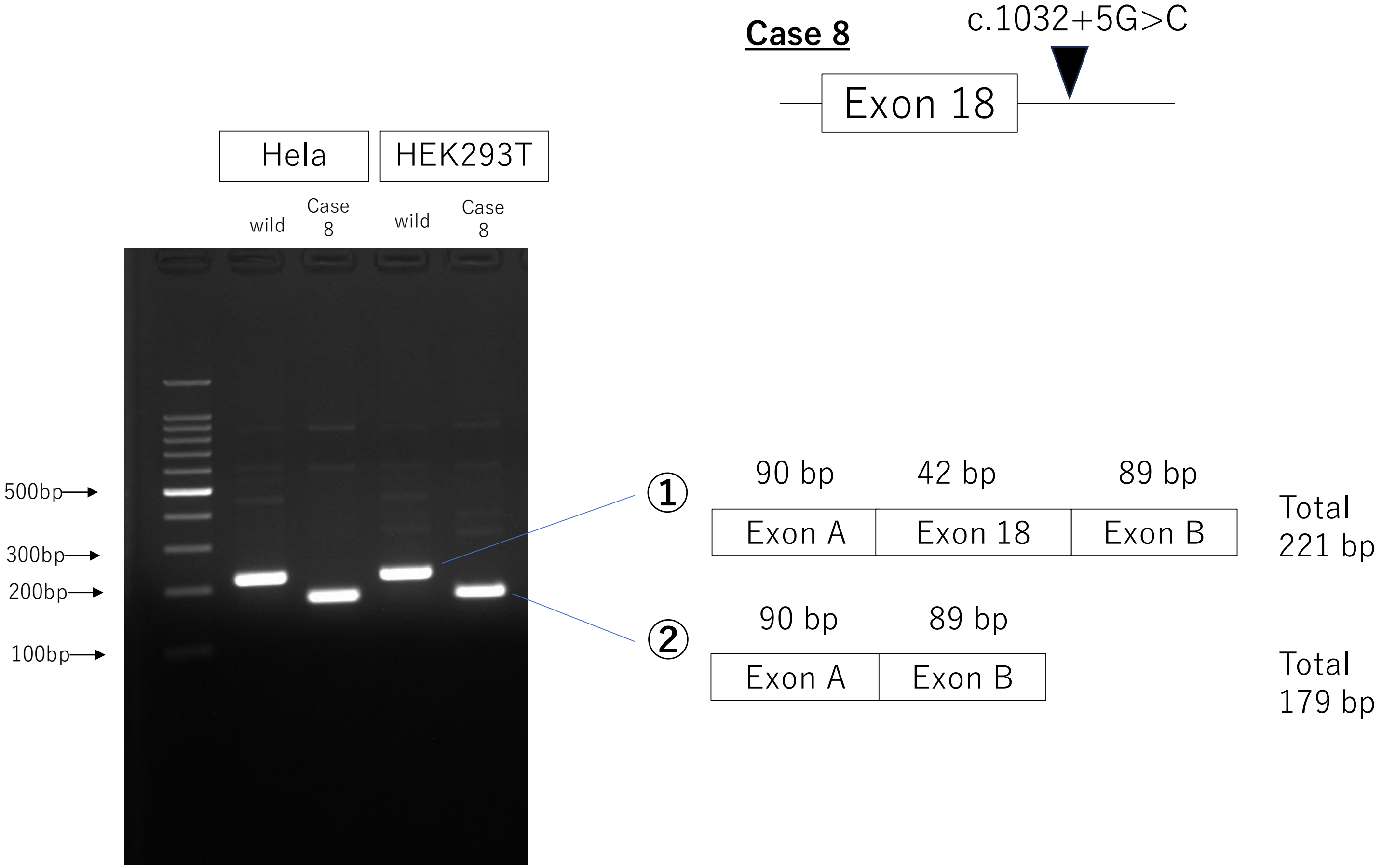
Case 8
c.1032+5G>C
Exon 18
Hela
HEK293T
wild
Case 8
wild
Case 8
500bp
300bp
200bp
100bp
①
90 bp
42 bp
89 bp
Exon A
Exon 18
Exon B
Total 221 bp
②
90 bp
89 bp
Exon A
Exon B
Total 179 bp

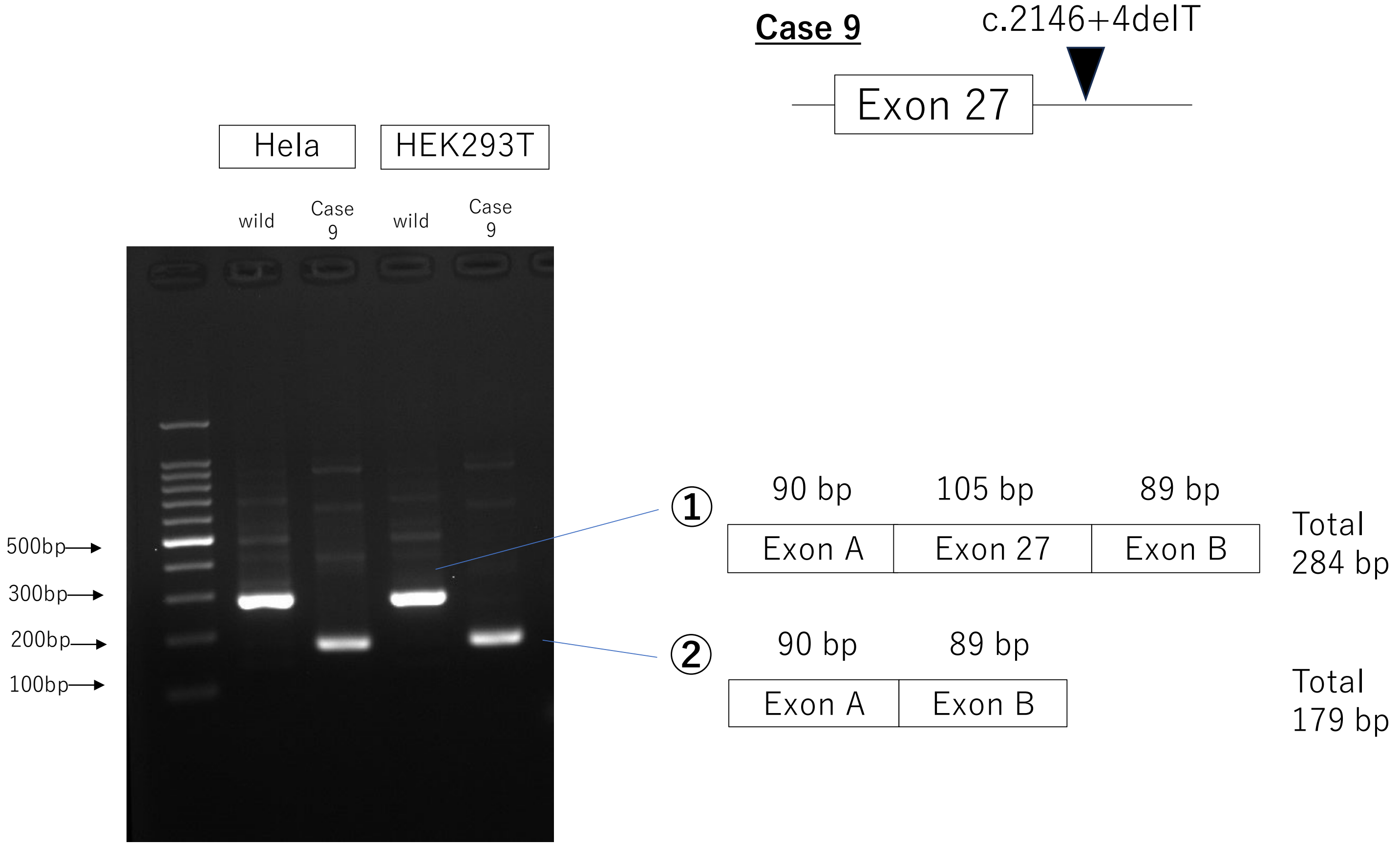
Case 9
c.2146+4delT
Exon 27
Hela
HEK293T
wild
Case 9
wild
Case 9
500bp
300bp
200bp
100bp
①
90 bp
105 bp
89 bp
Exon A
Exon 27
Exon B
Total 284 bp
②
90 bp
89 bp
Exon A
Exon B
Total 179 bp

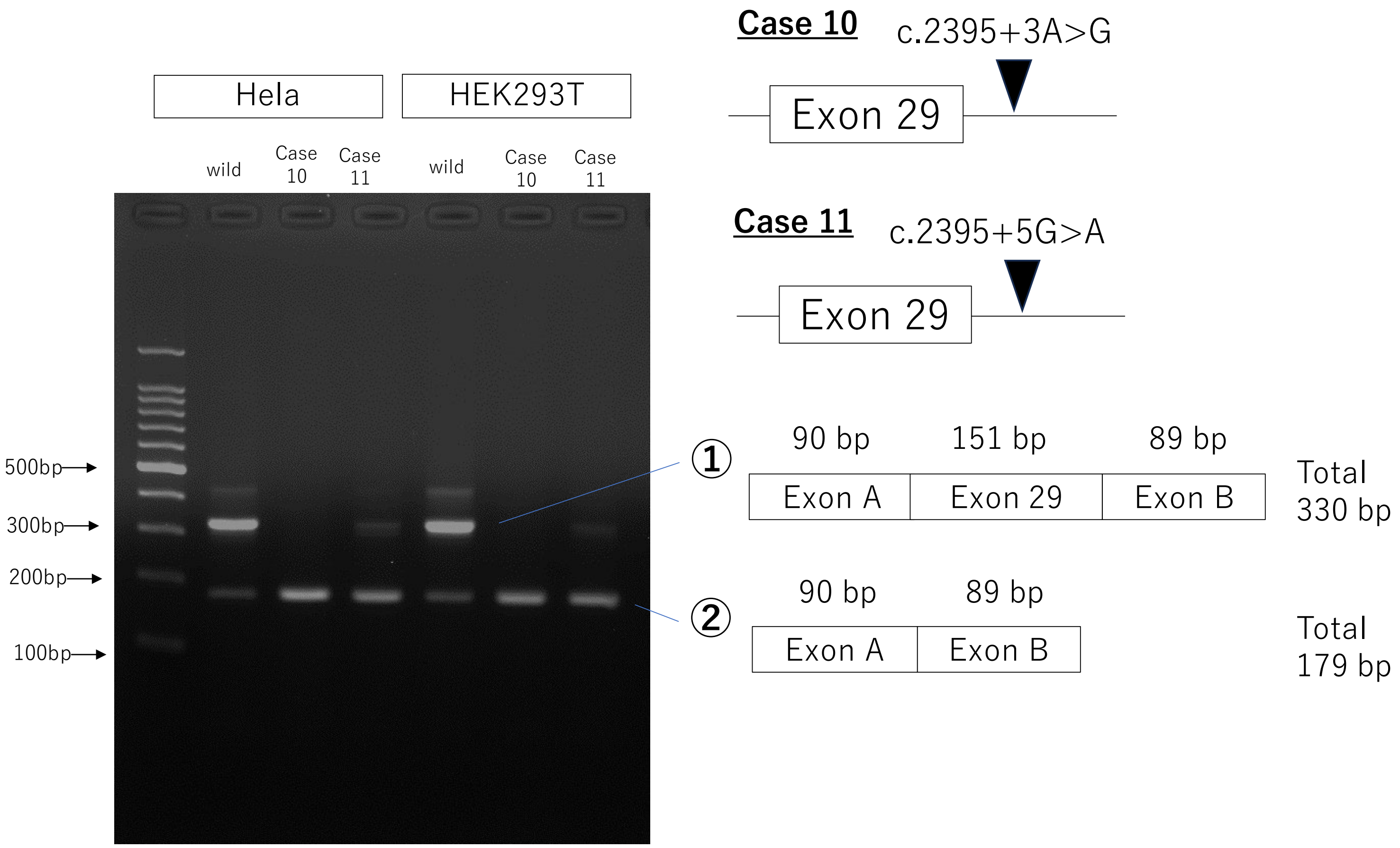
Hela
HEK293T
wild
Case 10
Case 11
wild
Case 10
Case 11
500bp
300bp
200bp
100bp
Case 10
c.2395+3A>G
Exon 29
Case 11
c.2395+5G>A
Exon 29
①
90 bp
151 bp
89 bp
Exon A
Exon 29
Exon B
Total 330 bp
②
90 bp
89 bp
Exon A
Exon B
Total 179 bp
